# Managing Post COVID-19 Patient with Breathlessness

**DOI:** 10.1155/2022/7512400

**Published:** 2022-08-04

**Authors:** Yen Shen Wong, Muhammad Amin Ibrahim, Mohd Arif Mohd Zim, Mohd Fauzi Abdul Rani

**Affiliations:** Faculty of Medicine, Universiti Teknologi MARA (UiTM), Sungai Buloh, Selangor, Malaysia

## Abstract

**Background:**

Chronic respiratory disease may be associated with severity of coronavirus disease 2019 (COVID-19) infection. We review a case of chronic obstructive pulmonary disease (COPD) patient who developed acute breathlessness post COVID-19 infection, also focusing on the diagnostic approach.

**Case:**

A 69-year-old gentleman with background history of COPD GOLD D and ischemic heart disease was admitted with severe COVID-19 infection. He required high-flow nasal cannula upon presentation. A computed tomography pulmonary angiography (CTPA) thorax at day 10 of illness revealed moderate organizing pneumonia (OP) with emphysematous changes, without pulmonary embolism. He received oral baricitinib and intravenous methylprednisolone for 3 days, which was then followed by tapering prednisolone starting dose of 1 mg/kg/day (60 mg/day) with reduction of 10 mg prednisolone every 3 days. COPD pharmacotherapy was optimized with early utilization of dual bronchodilators and inhaled corticosteroid was withheld. He underwent inpatient pulmonary rehabilitation and was discharged with home oxygen therapy. Unfortunately, he was re-admitted after 2 weeks with shortness of breath and fever for 3 days. Blood results revealed leucocytosis with raised C-reactive protein. A repeat CTPA showed increase reticulations and crazy paving pattern with reduction in lung volume. Multidisciplinary team discussion concluded it as interstitial pneumonia with COVID-19 OP and fibrosis progression. Prednisolone was stopped and he responded well with antibiotics. A follow-up at 3 months post COVID-19 infection showed improvement of clinical symptoms with radiological resolution of ground glass changes.

**Conclusion:**

Corticosteroid inhaler should be cautioned in this case, in view of recent pneumonia and non-elevated serum eosinophil count. These groups of patients should be closely followed up to unmask interstitial lung disease that may present prior to COVID-19 and worsen post-infection. Optimizing pre-existing medical conditions should be the paramount intervention.

## 1. Introduction

Most coronavirus disease 2019 (COVID-19) survivors return to their baseline state of health after acute COVID-19 infection, but a proportion of patients experience worsening respiratory symptoms. The complex pathophysiology that happens post COVID-19 infection leads to a wide range of differential diagnoses for COVID-19 survival who presented again with breathlessness. This could be related to their own pulmonary commodities, previous undiagnosed interstitial lung disease, or post COVID-19 infection complications such as pulmonary embolism, organizing pneumonia, and secondary bacterial infection. In this literature, we review a case of chronic obstructive pulmonary disease (COPD) patient who developed acute breathlessness post COVID-19 infection, also focusing on the diagnostic approach.

## 2. Case

A 69-year-old gentleman with 120-pack-year smoking history was admitted with RT PCR-confirmed COVID-19 infection. He has background history of diabetes, hypertension, ischemic heart disease, and COPD GOLD D. He was on tiotropium and olodaterol inhalers, tablet aspirin, clopidogrel, atorvastatin, and basal bolus insulin injection.

**Figure 1:**
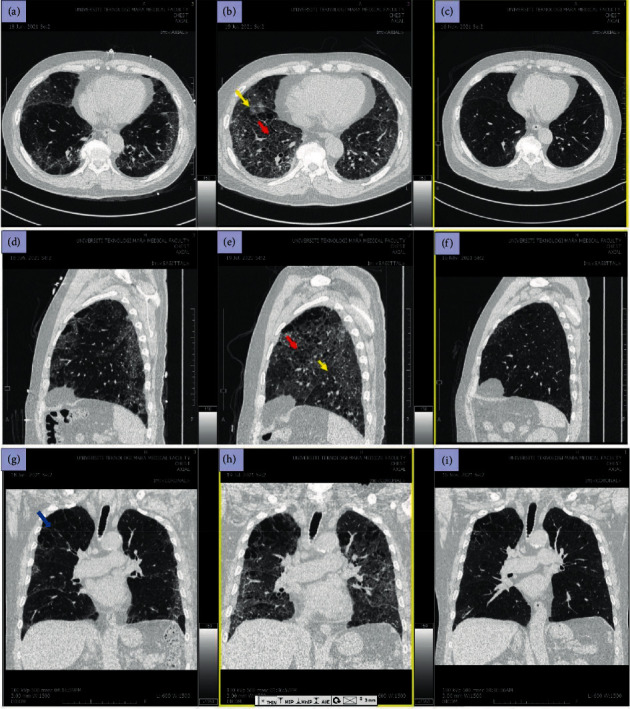
The sequences of CT thorax at day 10 of COVID-19 illness (a–c) showed moderate GGO with organizing pneumonia in the background of emphysematous lung (blue arrow). Second CT thorax at day 42 post COVID-19 infection (d–f) showed worsening bilateral ground glass opacity with reticulations and crazy paving pattern (red arrow) most prominent at bilateral lower lobes. There is also reduction in lung volume with displaced fissures (yellow arrow). Third CT thorax at 5 months post COVID-19 infection (g–i) showed significant improvement of GGO with residual reticulations in apical region and lung bases.

During the 1^st^ admission for COVID-19 infection, he presented with 3-day history of cough and shortness of breath. Clinically, he was tachypnoeic with RR 28 bpm. Arterial blood gas showed type 1 respiratory failure and he required high-flow nasal cannula FiO_2_ 0.6 60 L/min. A COVID-19 polymerase chain reaction (PCR) test was positive with raised CRP 20 mg/L. Otherwise, white blood cells (WBC) were normal (7 × 10^9^/L) and other blood tests were unremarkable. Chest radiograph showed bilateral lower zone ground glass opacity. A computed tomography pulmonary angiography (CTPA) thorax on day 10 of illness revealed moderate organizing pneumonia with a background of emphysematous changes and no pulmonary embolism (Figures 1(a), 1(b), and 1(c)).

He was treated with oral baricitinib and intravenous methylprednisolone for 3 days which was then changed to tapering dose of prednisolone with starting dose of 1 mg/kg/day (60 mg/day) and reduction of 10 mg prednisolone every three days. He underwent inpatient pulmonary rehabilitation and was discharged after 22 days of admission with short-term oxygen therapy.

He presented again after two weeks with three-day history of cough and shortness of breath. Clinically, he was febrile with temperature of 38°C. He appeared tachypnoeic with SpO2 92% under room air. Lung examination revealed minimal bi-basal fine crepitations without audible wheeze. There was no pedal oedema and jugular venous pressure was not raised. Blood investigation showed leucocytosis with WBC 16 × 10^9^/L and raised CRP 45 mg/L. Other blood investigations including renal profile and liver function test were normal. Sputum and blood culture were negative. A repeated CTPA thorax showed worsening ground glass opacity with reticulations and crazy paving pattern. There was also reduction in lung volume with displaced fissures. Otherwise, there was no organizing pneumonia or pulmonary embolism (Figures 1(d), 1(e), and 1(f)).

Multidisciplinary team discussion concluded it as post COVID-19 infection with progressive fibrosis and concomitant secondary bacterial infection. He was treated for hospital-acquired pneumonia with intravenous piperacillin/tazobactam 4.5 g QID. The prednisolone was stopped due to an active infection. Bronchoscopy was not performed as he responded well with the antibiotic therapy. He was discharged after 1 week with long-acting beta_2_-agonist (LABA) plus long-acting muscarinic antagonist (LAMA) combination inhaler and underwent outpatient pulmonary rehabilitation.

A follow-up at 3 months post COVID-19 infection showed improvement of clinical symptoms and radiological resolution of crazy paving pattern (Figures 1(g), 1(h), and 1(i)). His full lung function at 3 months post COVID-19 infection revealed restrictive ventilatory defect with forced expiratory volume in 1 second (FEV_1_) of 1.67 L (72% predicted), forced vital capacity (FVC) of 1.74 L (64% predicted), ratio FEV1/FVC_1_ 0.96, total lung capacity (TLC) of 69% predicted, and diffusing capacity of carbon monoxide (DLCO) of 59% predicted. In addition, impulse oscillometry (IOS) showed an elevated difference between resistance at 5 Hz and 20 Hz (R5-R20) of 0.13 kPa/L/S. The presence of restrictive ventilatory defect with IOS results supports the diagnosis of post COVID-19 interstitial lung disease with concomitant COPD with emphysema. At this juncture, we have diagnosed him with resolving interstitial lung disease (ILD) and therefore no definitive treatment for ILD was warranted.

## 3. Discussion

COVID-19 pneumonia-related dyspnoea is likely to improve over time but may have a protracted course, especially in our patient who has severe pulmonary involvement. The general treatment strategy should address the underlying reasons of dyspnoea, which is often multifactorial. In this case, the probable causes of dyspnoea include exacerbation of COPD, secondary bacteria infection, and post COVID-19 pumonary fibrosis. We have optimized COPD pharmacotherapy with early utilization of dual bronchodilators based on the severity of the symptoms. Inhaled corticosteroid inhaler should be cautioned in this case, in view of recent pneumonia [1, 2] and non-elevated serum eosinophil count [3]. Furthermore, it should not be used as a part of triple therapy for the initial pharmacotherapy option. Inhaled corticosteroids have been recently investigated for the potential treatment of hyperinflammation in COVID-19 infection; however, current data are insufficient to support the efficacy in treating acute COVID-19 infection [4–6].

We prescribed home oxygen therapy for this patient in view of severe hypoxaemia. There was doubtful recovery of the lung function as existing lung abnormalities were further impaired by the recent COVID-19 pneumonia. Elderly patients with pre-existing lung abnormality were associated with prolonged hospital admission and difficulty of weaning oxygen [7]. American Thoracic Society and British Thoracic Society (BTS) guidelines recommended severe hypoxaemia to be defined as PaO2 60 mmHg or 7.3 kPa, or SpO2 88% on room air in patients with chronic lung disease [8, 9]. However, a recent study suggested 92% on maximum of 3 L/min oxygen flow as the criteria for discharge with home oxygen for COVID-19 patients [10]. The study showed low mortality and re-admission rate in their cohort. Low threshold to prescribe home oxygen therapy in post COVID-19 was a reasonable approach to allow early discharge and preserve limited acute care bed.

Unfortunately, the patient was re-admitted to our centre with worsening symptoms despite dual bronchodilators and home oxygen therapy. This raised the question of the possibility of ongoing organizing pneumonia from resolving the COVID-19 infection and re-introduction of steroid treatment. In a retrospective study of 837 survivors of COVID-19, 5% had evidence of organizing pneumonia, and initiation of corticosteroid treatment resulted in improved symptoms, imaging, and function [11]. However, adopting a similar approach in this case should be cautioned as the patient presented with fever and repeat CT scan predominantly showed worsening ground glass changes with the absence of organizing pneumonia features. Furthermore, the CT scan findings should be interpreted with accompanying clinical contexts, as in this case, bacterial infection and patient did improve with broad-spectrum antibiotic. Prescribing prolonged steroid treatment without exclusion of infection would be detrimental and associated with unnecessary steroid toxicity.

Post COVID-19 pulmonary fibrosis is defined as the presence of persistent fibrotic changes identified on follow-up CT scans and associated with functional impairment [12]. The prevalence was between 9% and 85% [13, 14], and these wide variations were mainly attributed to the timing of the assessment [15]. The risk factors for progression of post COVID-19 pulmonary fibrosis were related to patient factors and COVID-19 severity, namely, in the present case, age >50 years, significant smoking history, chronic obstructive pulmonary disease with emphysema, severe COVID-19 pneumonia, and the use of non-invasive ventilation. Furthermore, chronic obstructive pulmonary disease was the only comorbidity associated with post COVID-19 pulmonary fibrosis [15].

Meta-analysis of post COVID-19 infection demonstrated progression of acute viral pneumonitis to parenchyma lung damage and fibrotic changes within three to six months [16]. However, none of the fibrotic-like changes were described as end-stage lung fibrosis changes [17]. Majority of restrictive impairment observed within one and three months among post COVID-19 patients had resolved by 12 months [16]. However, the proportion of patients with abnormal gas transfer remained elevated at the same period. None of these patients were subjected to specific treatments or interventions.

The definite treatment for post COVID-19 pulmonary fibrosis is not completely determined yet. An observational study of early corticosteroid treatment has reported rapid and significant improvement in lung volume and gas transfer in persistent post COVID-19 interstitial lung disease patients (ILD) [11]. However, there was lack of control group for comparison and the improvements may reflect ongoing natural lung recovery post COVID-19 infection. Anti-fibrotic showed potential inhibition of acute lung injury [18] and anti-inflammatory properties [18–20]; however, its safety profile, efficacy, subgroups of patients who would benefit, and the timing for initiation of treatment have not yet been established for COVID-19 patients.

Patients with ILD and a pre-existing risk factor for lung fibrosis as described in our case should be closely followed up after COVID-19 infection for progression of ILD. BTS recommended comprehensive assessments of symptoms, lung physiology, and imaging at 3 months while further serial assessments at 6 months and 12 months were warranted to differentiate those who have resolving and progressive ILD [21]. At the present juncture, we have diagnosed him with resolving ILD and therefore no definitive treatment for ILD was warranted. However, we remain cautious of the risk of ILD progression and therefore subsequent follow-up is required as per BTS recommendation.

## 4. Conclusion

The causes of dyspnoea in post COVID-19 patient are often multifactorial. Comprehensive assessments of symptoms, lung physiology, and chest imaging will navigate the differential diagnosis. These groups of patients should be closely followed up to unmask interstitial lung disease that may present prior to COVID-19 and worsen post-infection. Optimizing pre-existing medical conditions should be the paramount intervention while specific treatment for COVID-19 ILD remains experimental at this moment.

## Data Availability

The data used to support the findings of this study are included within the article.
